# The global landscape of intron retentions in lung adenocarcinoma

**DOI:** 10.1186/1755-8794-7-15

**Published:** 2014-03-20

**Authors:** Qu Zhang, Hua Li, Hong Jin, Huibiao Tan, Jun Zhang, Sitong Sheng

**Affiliations:** 1 Department of Human Evolutionary Biology, Harvard University, Cambridge, MA 02138, USA; 2 HYK High-throughput Biotechnology Institute, 4/F, Building #11, Software Park, 2nd Central Keji Rd, Hi-Tech Industrial Park, Shenzhen 518060, China; 3 Department of Surgery, Shanghai Institute of Digestive Surgery, Ruijin Hospital, Shanghai Jiaotong University School of Medicine, No.197 Ruijin 2nd Road, Shanghai 200025, China; 4 School of Bioscience and Bioengineering, South China University of Technology, Guangzhou Higher Education Mega Center, Guangzhou 510006, China; 5 College of Life Sciences, Shenzhen University, Shenzhen 518060, China

**Keywords:** Intron retentions, RNA-Seq, Lung adenocarcinoma, Gene expression, Nonsense mediated decay

## Abstract

**Background:**

The transcriptome complexity in an organism can be achieved by alternative splicing of precursor messenger RNAs. It has been revealed that alternations in mRNA splicing play an important role in a number of diseases including human cancers.

**Methods:**

In this study, we exploited whole transcriptome sequencing data from five lung adenocarcinoma tissues and their matched normal tissues to interrogate intron retention, a less studied alternative splicing form which has profound structural and functional consequence by modifying open reading frame or inserting premature stop codons.

**Results:**

Abundant intron retention events were found in both tumor and normal tissues, and 2,340 and 1,422 genes only contain tumor-specific retentions and normal-specific retentions, respectively. Combined with gene expression analysis, we showed that genes with tumor-specific retentions tend to be over-expressed in tumors, and the abundance of intron retention within genes is negatively related with gene expression, indicating the action of nonsense mediated decay. Further functional analysis demonstrated that genes with tumor-specific retentions include known lung cancer driver genes and are found enriched in pathways important in carcinogenesis.

**Conclusions:**

We hypothesize that intron retentions and consequent nonsense mediated decay may collectively counteract the over-expression of genes promoting cancer development. Identification of genes with tumor-specific retentions may also help develop targeted therapies.

## Background

As one of the leading causes of cancer-related mortality in the world, lung cancer accounts for approximately 12 percent of all cancer incidences and 17.6 percent of cancer deaths [1,2]. Of them, lung adenocarcinoma accounts for more than 500,000 deaths per year worldwide and is the most common subtype of non-small cell lung cancer [3]. Although the underlying mechanism of lung adenocarcinoma is still under investigation, studies showed that recurrent mutations in the epidermal growth factor receptor (*EGFR*) and the anaplastic lymphoma kinase (*ALK*) fusions could change the efficacy of treatment for patients with lung adenocarcinoma [4-8]. Genetic modifications in other genes, including targeted mutations in *BRAF*, *AKT1*, *ERBB2* and *PIK3CA*, as well as *ROS1*- and *RET*-involved fusions, may also affect cancer therapy [9]. In addition, a recent study has found frequent copy number changes in *NKX2-1*, *TERT*, *PTEN*, *MDM2*, *CCND1*, and *MYC* in lung adenocarcinoma [3], highlighting the role of various types of genetic alternations in carcinogenesis.

Alternative splicing in multiple-exon genes is prevalent in eukaryotes and it is actively involved in development, cell differentiation and disease. Approximately 90% of multi-exon human genes have splicing variants in different tissues and cell lines [10,11]. Intron retention, or the maintenance of an intron in a mature mRNA transcript, is a less common type of alternative splicing [12] and can have large functional consequence by introducing premature mutations to the mature transcript. Although the impact of intron retentions has been less acknowledged, a recent report suggests that intron retention is one of the most predominant splice events in three breast cancer subtypes [13], and the retention of intron 4 in the wild-type cholecystokinin type 2 (*CCK*_*2*_) receptor shows elevated expression associated with increased tumor growth in a few cancers [14].

The emergence of high-throughput sequencing technologies in the past few years has provided a new platform to perform large-scale transcriptome profiling at an affordable cost. Based on high-throughput sequencing, RNA-Seq can precisely measure mRNA expression and characterize gene isoforms [15,16], and is commonly used to identify somatic mutations [17,18], differentially expressed genes [19], fusion genes in tumor tissue [20-22], and allele-specific expression [23,24]. Here in the present study, we exploited the rich information in RNA-seq data to investigate the potential role of intron retentions in lung adenocarcinoma. Using tumor and matched normal samples, we systematically identified genes with tumor-specific intron retentions. Further investigation suggests a potential protective role of intron retentions in carcinogenesis through the action of nonsense mediated decay (NMD).

## Methods

### Transcriptome dataset

Transcriptome sequencing data from five lung adenocarcinoma and their paired adjacent normal tissue specimens [17] were downloaded from European Nucleotide Archive (ENA, http://www.ebi.ac.uk/ena/), using the accession number ERP001058. Reads from five patients (LC1, LC5, LC10, LC11, and LC12) were used in this study, and as described in the original study, all protocols were approved by the Institutional Review Board of Seoul National University Hospital (Approval # C-1111-102-387) and Seoul St. Mary’s Hospital (Approval # KC11TISI0678). 101-bp paired-end reads were generated by Illumina Hiseq 2000 sequencer for each sample.

### Exon-intron junction data

To extract exon-intron junction sequences, human exon information was first downloaded from Ensembl database (release 69) [25]. To assign exon-intron junction unambiguously, intersecting exons were excluded, resulting in 164,500 non-overlapping exons. Then exon-intron junctions were then determined and 101-bp sequences were extended in each direction for future mapping.

### Identification of intron retentions

A strategy of two rounds of short read mapping was adopted to identify retention reads. Bowtie2 [26] was first used to align RNA-Seq reads to human cDNA sequences (Ensembl release 69), with seed length as 20-bp. Unmapped reads were then extracted and mapped to exon-intron junctions by Bowtie2, using the same parameter above. Uniquely mapped reads with a minimum quality score of 30 that cover a 20-bp region centered on the exon-intron junction site and have at most two mismatches within this region were defined as retention reads. A common tumor-specific retention (TSR) was defined as an exon-intron junction that was supported by retention reads from at least two tumor samples and no retention read could be found in any normal sample. Normal-specific retention (NSR) was similarly defined. For each candidate TSR, we estimated its relative abundance by recording the number of reads that covered the junction position in the initial alignment, and reads with insertion or deletions were excluded. Then for a gene, the relative abundance of intron retentions was calculated as:

$$R_{i}=\frac{\sum_{i=1}^{n}\mathrm{retention}\;\mathrm{read}\;\mathrm{of}\;\mathrm{gene}\;i}{\sum_{i=1}^{n}\mathrm{retention}\;\mathrm{reads}\;\mathrm{of}\;\mathrm{gene}\;i+\sum_{i=1}^{n}\mathrm{reads}\;\mathrm{mapped}\;\mathrm{to}\;\mathrm{cDNA}\;\mathrm{for}\;\mathrm{gene}\;i}$$

where R_*i*_ is the retention abundance for gene *i*.

### Identification of differentially expressed genes (DEGs)

Gene expression was first calculated by using the RSEM program [27], which effectively uses ambiguously-mapping reads to estimate expression abundance. Next, EdgeR package was used to normalize the data by trimmed mean of M values (TMM) and identify differentially expressed genes [28]. Genes at low expression level (≤ 1 transcript per million reads, TPM) were excluded and DEGs were defined as genes with a *p*-value < 0.05 after Benjamini-Hochberg adjustment [29].

### Identification of tumor-specific variants (TSVs)

Variants in tumor samples were first identified by SAMtools [30] for each patient, and only variants supported by at least three reads with base quality ≥20 were retained. Positions of those variants were then examined in normal samples to make sure they were also covered by reads from the corresponding normal samples and they were not variable in normal samples.

### Functional enrichment and pathway analysis

Gene ontology (GO) [31] information for query genes was assigned using bioconductor (http://www.bioconductor.org) package “org.Hs.eg.db”. Enrichment tests were performed by assuming a hypergeometric distribution using “topGO” package [32]. KEGG (Kyoto Encyclopedia of Genes and Genomes) database [33] was used to retrieve pathway annotation information, and Fisher’s exact test was performed to evaluate the enrichment of a pathway. Multiple test correction was conducted using Benjamini-Hochberg method.

## Results

### Summary statistics of human intron retentions

The data used in this study were whole transcriptome sequencing from tumor and adjacent normal tissues of five patients with lung adenocarcinoma, containing approximately 665 million short reads produced by Illumina HiSeq2000 sequencer, about 67 million per sample (Additional file 1: Table S1). Using Bowtie 2 aligner, about 647 million reads (~97%) can be mapped to human cDNAs. The remaining 18 million unmapped reads were further aligned to exon-intron junctions to identify potential intron retention events. On average, 67,466 and 63,297 retention events were found in each tumor and normal sample, respectively, with ~36,865 retentions in common (Additional file 1: Table S1).

### Genes with intron retentions

We next sought to identify genes with tumor-specific intron retentions (TSRs). A TSR is an exon-intron junction that has been covered by retention reads in at least two tumor samples but none of normal samples. As a result, 4,099 TSRs were found by applying above criteria (Table 1), compared to 2,437 normal-specific intron retentions (NSRs). These tissue-specific intron retentions were further mapped to genes, resulting in 2,983 genes with TSRs and 1,991 genes with NSRs, respectively. Of these genes, 500 have both TSRs and NSRs, suggesting they may present different intron retention patterns in tumor and normal samples. For the remaining 2,483 genes only containing TSRs (tumor-specific retention genes, TSRGs, Additional file 2: Table S2), it is possible that some genes may not be expressed in the normal samples and thus lead to biased result. To exclude this possibility, we assessed the expression pattern of TSRGs and retained 2,340 genes that were expressed (>1 TPM) in all tumors and normal controls under investigation. Among them, 576 genes have more than one TSR. Comparatively, 1,422 expressed normal-specific retention genes (NSRGs) were found, and 220 have more than one NSR (Table 1).

**Table 1:** Summary statistics for intron retentions in normal and tumor samples

	**Normal**	**Tumor**
Group-specific retention (GSR)^a^	2437	4099
Genes with GSR^b^	1991	2983
Group-specific genes^c^	1491	2483
Group-specific genes (TPM > 1)^d^	1422	2340
Group-specific genes (TPM > 1 and GSR > 1)^e^	220	576

^a^Intron retentions only found in one group (TSR or NSR).

^b^Genes with TSR or NSR.

^c^Genes with TSR but not NSR or genes with NSR but not TSR.

^d^Group-specific genes that are expressed more than one transcript per million reads (TPM).

^e^Group-specific genes that are expressed more than one transcript per million reads (TPM) and have more than one GSR.

### Characterization of retained Introns

**Figure 1:**
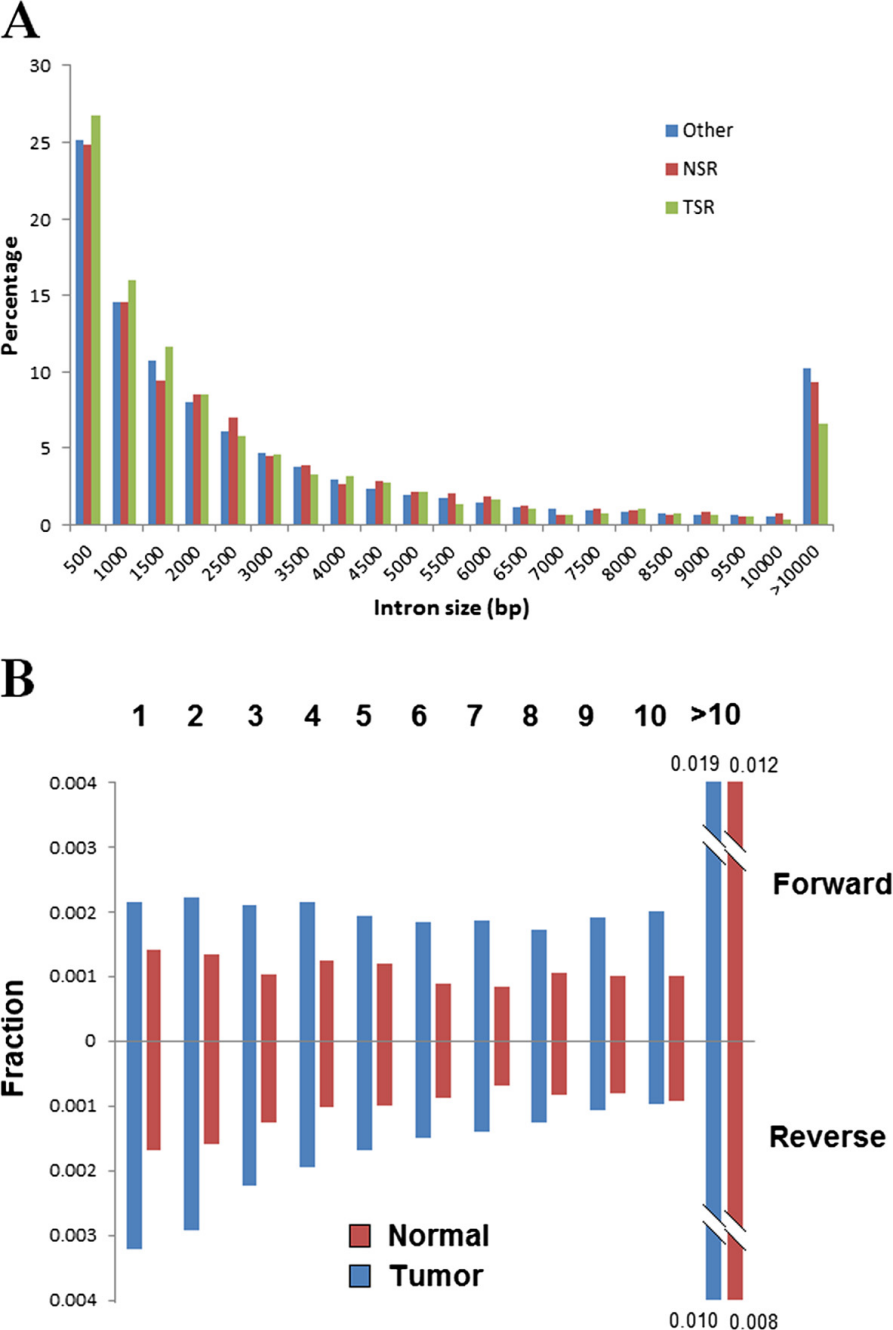
**Genomic features of retained introns. A**. The size distribution of introns in different classes. The bin size is 500 bp, and introns larger than 10,000 bp were included in one bin. The distribution showed tumor-specific retained introns are larger than other introns (see text). TSR: tumor-specific retained introns; NSR: normal-specific retained introns; Other: non-retained introns. **B**. The percentage of retained introns in different orders. Forward order is showed above the X-axis, and reverse order is showed under the X-axis. The histogram for introns after ten was truncated to fit the plot. Tumor and normal samples are labeled by blue and darkred.

It has been proposed that in vertebrates, shorter introns have a higher chance to be retained [34,35]. In order to examine whether it is the case here, we compared the size distribution of retained introns and non-retained ones (Figure 1), and found that tumor-specific retained introns are significantly shorter than non-retained introns (1,293 bp versus 1,483 bp, median size, *P*-value = 4.8 × 10^−7^, Wilconxon ranksum test). However, no such pattern was found for normal-specific retained introns (1,570 bp versus 1,483 bp, *P*-value = 0.81). We also found that the position of retained introns are not evenly distributed in the transcripts, and the last introns are most likely to be retained in both tumor and normal samples, but no obvious pattern can be found regarding the forward order of introns (Figure 1).

### Gene expression abundance and intron retention abundance

**Figure 2:**
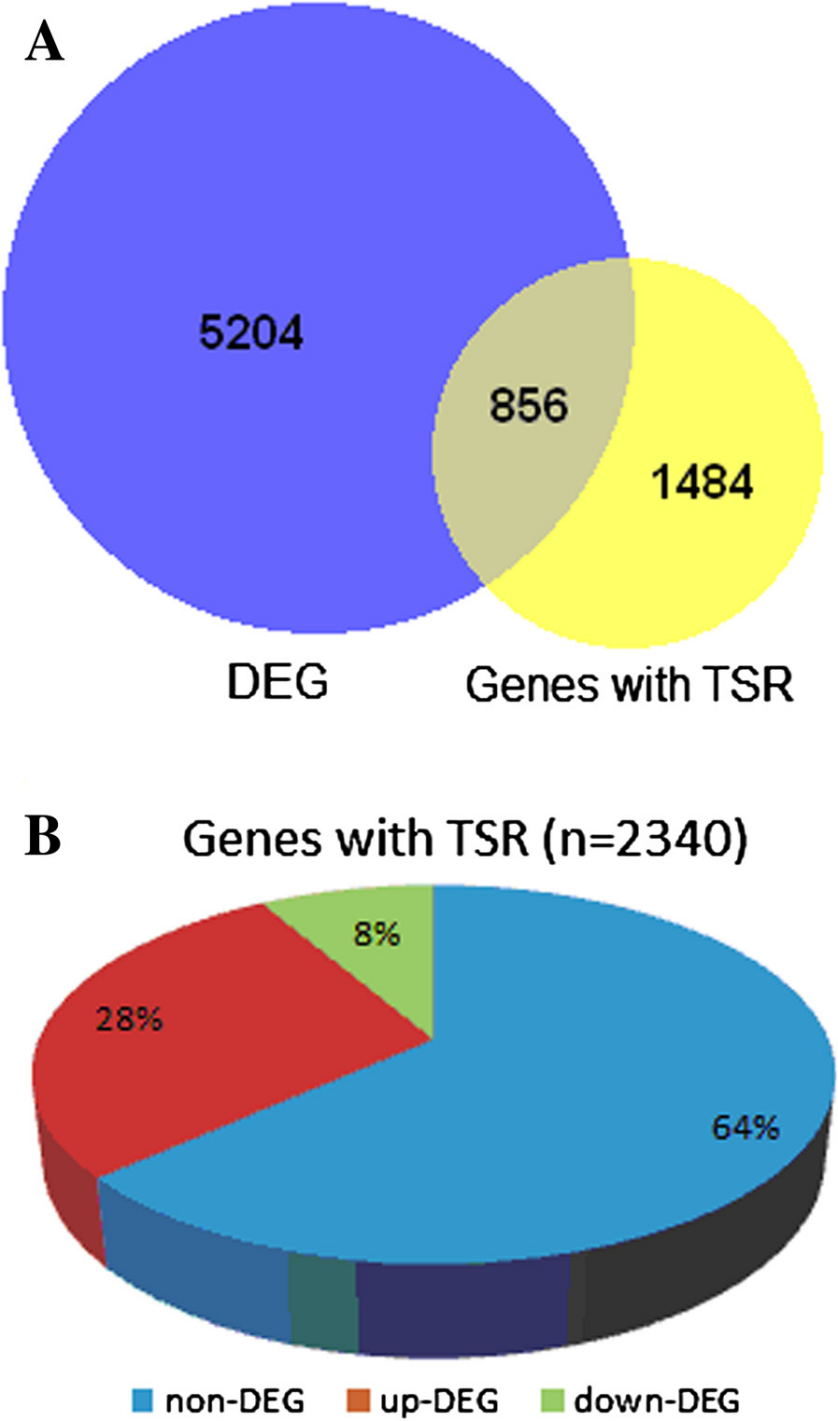
**Overrepresentation of genes with tumor-specific retention (TSR) in differentially expressed genes (DEGs) between tumor and normal samples. A**: Venn diagram of DEG and genes with TSR. **B**: pie chart of the expression pattern in tumor and normal tissues for genes with TSR. A majority of DEGs are up-regulated in tumors. Non-DEG: genes without differentially expression in tumor and normal tissues; up-DEG: differentially expressed genes up-regulated in tumor tissues; down-DEG: differentially expressed genes up-regulated in normal tissues.

Another question of interest is the relationship between intron retentions and the expression level of genes. To investigate it, we first used RSEM program to estimate gene expression and EdgeR package in bioconductor to identify genes that were differentially expressed between tumors and normal samples. In total, 6,060 differentially expressed genes (DEGs) were identified with a *P*-value < 0.05 (after Benjamini-Hochberg correction). Of them, 856 (~14%) genes were TSRGs (Figure 2), which were significantly enriched (*P*-value = 1.2 × 10^−9^, Fisher’s exact test, FET). Additionally, when only considering 576 TSRGs with multiple retentions, we found a more prominent overrepresentation of DEGs in this set (261 DEGs, *P*-value = 3.4 × 10^−13^, FET). Interestingly, a majority of TSRGs (659 of 856, 77%) are up-regulated in tumor samples, which is again highly unexpected (*P*-value < 2.2 × 10^−16^, binomial test). This skewness is more substantial in TSRGs with multiple retentions (239 of 261, 92%). We also studied the relationship within tumor samples and a majority of genes (1474 of 2340, 63%) showed a positive correlation between the intron retention count and expression level, which is significantly deviated from the null expectation (50%, *P*-value < 2.2 × 10^−16^, binomial test) and is consistent with previous observation [35].

One possible explanation for the over-representation of intron retentions in tumors is the inhibition of nonsense mediated decay (NMD), which degrades transcripts with pre-mature codons [36] and is reported to be inhibited in tumor microenvironment [37]. Therefore we investigated the expression pattern of 136 genes involved in NMD process (Additional file 3: Table S3). Among them, only three genes (*CTIF*, *FAU*, and *RPS28*) are significantly down-regulated in tumors, implying NMD may not be inhibited in lung adenocarcinoma and thus cannot explain the large amount of intron retentions in tumors.

It should be noted that the observed correlation could be simply explained by that retentions in over-expressed genes are preferentially identified due to their abundance. Therefore we estimated the intron retention abundance in each TSRG. The mean and median abundance of transcripts with intron retentions for a TSRG in tumor samples are 18% and 5%, consistent with previous observation that intron retentions comprise a minor fraction of splicing forms [10]. Furthermore, we found that up-regulated TSRGs have lower percentage of intron retentions compared with down-regulated genes (4.5% versus 6.7%, median percentage, *P*-value < 2.2 × 10^−16^, Wilconxon ranksum test). As transcripts with premature stop codons tend to be degraded by NMD [36], the low level of intron retentions in up-regulated TSRGs and vice versa may suggest the presence of NMD. To validate this, we categorized 2,340 commonly expressed TSRGs as genes with in-frame retention (659) and genes with frame-shift retention (1681), and compared their expression level as well as retention abundance. Genes with in-frame retentions have higher expression than those with frame-shift retentions (1581 versus 1356, mean TPM), but it was not statistically significant (*P*-value = 0.4561, Wilconxon ranksum test). The retention level in genes with in-frame retentions is significantly higher compared with genes with frame-shift retentions (6.3% versus 4.8%, median percentage, *P*-value = 4.0 × 10^−12^, Wilconxon ranksum test), confirming NMD is active in tumor samples.

### Functional analysis of TSRGs

To understand the potential functional relevance of those 2,340 TSRGs, we further performed gene ontology analysis. Of 19,520 expressed genes, TSRGs were found to be enriched for 36 GO molecular function terms, including binding terms and kinase activity terms, as well as four cellular component terms (Additional file 4: Table S4). For the subset of TSRGs with multiple TSRs, six biological process terms, 14 molecular function terms and five cellular component terms were found overrepresented (Table 2). Interestingly, several collagen-related terms were found, such as GO0030199 (collagen fibril organization), GO0005583 (fibrillar collagen) and GO0005581 (collagen). As the most abundant proteins in the extracellular matrix, collagen plays an essential role to prevent tumor cells metastasizing to various sites throughout the body [38]. The abundant intron retentions in collagen genes may facilitate their degradation during tumor metastasis.

**Table 2:** Enriched gene ontology categories in genes with multiple TSRs

**Category**^**a**^	**Term**	**Corrected**^**b**^	**Class**^**c**^
hsa05322	Systemic lupus erythematosus	4.99E-08	KEGG
GO:0005583	Fibrillar collagen	0.00163	GO:CC
hsa03010	Ribosome	1.74E-03	KEGG
GO:0005201	Extracellular matrix structural constitu…	0.006651	GO:MF
GO:0007411	Axon guidance	0.009976	GO:BP
GO:0043062	Extracellular structure organization	0.009976	GO:BP
GO:0030199	Collagen fibril organization	0.009976	GO:BP
hsa00970	Aminoacyl-tRNA biosynthesis	1.03E-02	KEGG
hsa04370	VEGF signaling pathway	1.03E-02	KEGG
GO:0048407	Platelet-derived growth factor binding	0.011402	GO:MF
GO:0044420	Extracellular matrix part	0.015466	GO:CC
GO:0005581	Collagen	0.015466	GO:CC
GO:0030198	Extracellular matrix organization	0.020783	GO:BP
GO:0006935	Chemotaxis	0.022168	GO:BP
GO:0042330	Taxis	0.022168	GO:BP
GO:0031256	Leading edge membrane	0.028215	GO:CC
hsa04510	Focal adhesion	3.64E-02	KEGG
GO:0005578	Proteinaceous extracellular matrix	0.047652	GO:CC

^a^Categories with more than 1000 genes were removed.

^b^Fisher’s exact test after Benjamini-Hochberg correction.

^c^GO: gene ontology; KEGG: Kyoto Encyclopedia of Genes and Genomes; BP: biological process; MF: molecular function; CC: cellular component.

We also conducted pathway analysis for TSRGs and five pathways were overrepresented (Table 2), including the VEGF (vascular endothelial growth factor) signaling pathway (hsa04370). This signal pathway contains several key mediators of angiogenesis and lymphangiogenesis in tumor development [39], and is often found highly expressed in tumors [40]. Enriched intron retentions in these genes, again may activate the mRNA decay mechanism to offset the over-expression.

### Investigation of potential cause of intron retentions

One plausible reason for intron retentions is that mutations occurred on the intron splicing sites which change the splicing signal and thus result in an unspliced intron. To explore the prevalence of splicing mutations in tumors, we used SAMtools to identify single nucleotide variants in tumors, and then filtered ones also variable in the matched normal samples. In total, only 27 tumor-specific variants were found to modify the splicing signal (Additional file 5: Table S5). Considering the large number of tumor-specific intron retentions (4,099), it seems that somatic mutations on splicing sites may have a negligible role in causing intron retentions. We also investigated the expression level of several trans-acting splicing activators, including *Tra2*[41,42] and *RNPS1*[43], but none shows differential expression between tumors and normal samples.

### Intron retentions and tumor genes

By searching the COSMIC database (Catalogue of Somatic Mutations in Cancer, http://cancer.sanger.ac.uk/cancergenome/projects/cosmic/), we found TSRGs include a substantial number of tumor genes, and some are also represented in the Cancer Gene Census [44], which catalogues genes with mutations that have been causally implicated in cancer. Examples include *EGFR* (epidermal growth factor receptor), *KDR* (kinase insert domain receptor), *ATM* (ataxia telangiectasia mutated), and *ROS1* (c-ros oncogene 1, receptor tyrosine kinase). Furthermore, three genes were among the top 20 most frequently mutated genes in lung adenocarcinoma: *EGFR* (34%), *ATM* (5%) and *KDR* (5%). TSRG list in this study also targets other genes with a potential role in carcinogenesis, such as *MUC16* (mucin 16, cell surface associated), expression of which was found to correlate with clinical outcome in adenocarcinomas [45], as well as *RUNX1* (runt-related transcription factor 1), which binds to the core element of many enhancers and promoters and may have various roles in tumors [46,47]. A close investigation further found reads across six exon-intron junctions in *MUC16*, and the expression of *MUC16* is significantly elevated in tumors (*p*-value = 3.98 × 10^−13^ after Benjamini-Hochberg correction), but the abundance of intron retention is 3.4%, smaller than 4.5%, the median abundance of up-regulated TSRGs, implying the over-expression of *MUC16* in lung adenocarcinoma may be related to the below average intron retention level. Finally, we also prioritized a list of TSRGs which contain multiple frame-shift retentions and were significantly over-expressed in tumor samples (Additional file 6: Table S6). These genes include driver genes such as *EGFR*, *ROS1*, and *RUNX1*, thus functional studies on them should help understand the role of intron retentions in lung tumor development.

## Discussion

Recent large-scale efforts from Cancer Genome Atlas Research Network have resulted in lung cancer candidate genes with somatic mutations and copy number alternations [3,48]. However, variations at the mRNA level in these are not fully explored, though the diversity and functionality of tumor-specific transcripts have been highlighted [10,49,50]. Several processes could result in novel mRNA isoforms in tumors, including alterations in promoter usage, exon skipping, and splicing signals, which in consequence changes coding regions and the resulting proteins [51-53]. Thus it is essential to understand the contribution of cancer-related changes emerging at the stage of transcription. The rapid development of sequencing technology makes RNA-Seq a cost-effective way to characterize transcriptome and is therefore frequently used in biomedical studies. Here, we developed a bioinformatics pipeline that explores RNA-Seq data to identify intron retention events, a splicing form of less appreciation but be also important in cancer study [13,54], and further compared their spectrum between lung adenocarcinoma and matched normal tissues. A prevalence of intron retentions was found in carcinoma samples, and over-expressed TSRGs tend to have lower retention abundance compared with under-expressed genes.

One important issue in identifying intron retentions is to distinguish potential contaminations from genomic DNAs or precursor mRNAs during the library preparation process. In order to remove false positive calls caused by contamination, we applied a simple and straightforward filter that requires a candidate intron retention event to be presented in at least two tumor samples and not in any normal sample, or verse visa. If one sample is contaminated and contains false intron retentions, such retentions are not expected to be found in other samples; if multiple samples were contaminated, falsely called intron retentions would be found in both tumor and normal samples, which will also be removed by the filter. However, this filter also removes intron retentions occurred in individual samples, thus the total number of TSRs or NSRs should be even larger than reported here.

The nature of our bioinformatics pipeline determines that it may have limited power in detecting intron retentions in genes with low expression level, partially accounting for the enrichment of intron retentions in over-expressed genes. However, our pipeline also filtered genes with very low expression, the abundance of intron retentions in tumor samples thus cannot be simply explained by the expression bias. Additionally, when focusing on genes with abundant expression, a reverse pattern was demonstrated as the abundance of intron retention is negatively correlated with gene expression, which is likely the result of NMD. Since a substantial proportion of cancer driver genes are over-expressed in tumors, identified intron retentions in those up-regulated genes may suggest a biological role to neutralize over-expression in tumors.

With respect to the mechanism of somatic intron retentions, the most intuitive explanation is that somatic mutations occur at splicing sites and alternate the splicing signal, therefore those splicing sites could not be properly recognized. However, no enrichment of somatic mutations was observed in this dataset (less than 1% of TSRs have somatic mutations in the splicing sites). We also interrogated the expression pattern of several splicing activators, again, no obvious pattern was found. Alternatively, some studies showed that intron retention pattern is different among various tissues [55-57], suggesting other factors, such as cellular environment may also function in promoting the process of intron retention. In addition, the observation of smaller size of retained intron in tumors compared to that in normal samples or non-retained introns is intriguing. Although explanations have been proposed for short retained introns [35], the difference between normal and tumor samples remains unexplained. Future work is therefore necessary to better understand the pattern observed here.

Among genes with tumor-specific retentions, genes with known driver functions in cancer were rediscovered, including *EGFR*, *ROS1*, *ATM* and *KDR*. Additionally, other growth factor genes were also found with retained introns in tumor samples, such as *PDGFRB* (platelet-derived growth factor receptor, beta polypeptide), *TGFBI* (transforming growth factor, beta-induced), *EGF* (epidermal growth factor), *IGF2R* (insulin-like growth factor 2 receptor), and *ERBB2* (v-erb-b2 erythroblastic leukemia viral oncogene homolog 2), which are also involved in tumor evolution in various studies [58-62]. By detailed investigation, we found intron retentions within these genes all caused frame-shift changes, which tend to invoke NMD. It is well known that cancer driver genes, such as *EGFR*, are over-expressed or activated by mutations in tumors, further activating downstream pathways associated with cell growth and survival. Therefore intron retentions occurring in these over-expressed or highly mutable driver genes could be protective for the patient by triggering NMD, which in term reduces the expression level or copies of mutable mRNAs. Future validation studies and functional dissections, however, are still critical before we can draw the conclusion.

## Conclusions

At the moment of this analysis, only a few studies focus on systematically characterizing the global pattern and contribution of intron retentions in tumorigenesis [63]. Results in this study suggest a potential protective role of intron retentions in lung adenocarcinoma and may benefit further biomarker development. It would also be of interest to investigate the pattern of intron retentions in other cancer types.

## Consent

Written informed consent was obtained from patients in the original study and data is released for public use.

## Competing interest

The authors declare that they have no competing interest.

## Authors’ contribution

QZ and SS conceived the project. QZ, JZ, HJ, HT and JZ carried out the data analysis. QZ and SS drafted the manuscript. All authors read and approved the final manuscript.

## Pre-publication history

The pre-publication history for this paper can be accessed here:

http://www.biomedcentral.com/1755-8794/7/15/prepub

## Supplementary Material

Additional file 1: Table S1 Summary statistics for each sample. Click here for file

Additional file 2: Table S2 Genes with tumor-specific retentions and normal-specific retentions. Click here for file

Additional file 3: Table S3 Genes involved in nonsense mediated decay. Click here for file

Additional file 4: Table S4 Enriched gene ontology categories for genes with TSRs. Click here for file

Additional file 5: Table S5 Tumor-specific variants that changes the splicing signal. Click here for file

Additional file 6: Table S6 List of candidate tumor-specific retention genes for future functional study. Click here for file
